# Relationship between gamification elements and social and human factors using the simple additive weighting method

**DOI:** 10.1371/journal.pone.0320419

**Published:** 2025-04-16

**Authors:** Luz Marcela Restrepo-Tamayo, Gloria Piedad Gasca-Hurtado, Liliana Machuca-Villegas, Solbey Morillo-Puente

**Affiliations:** 1 Faculty of Engineering, Universidad de Medellín, Medellín, Colombia; 2 Faculty of Engineering, Universidad del Valle, Cali, Colombia; 3 Master of Science in Education program, Sabal University, Doral, Florida, United States of America; Autonomous University of Queretaro: Universidad Autonoma de Queretaro, MEXICO

## Abstract

Gamification is a strategy to stimulate social and human factors (SHF) that influence software development productivity. However, software development teams must improve their productivity to face the challenges of software development organizations. Traditionally, productivity analysis only includes technical factors. Literature shows the importance of SHFs in productivity. Furthermore, gamification elements can contribute to enhancing such factors to improve performance. Thus, to design strategies to enhance a specific SHF, it is essential to identify how gamification elements are related to these factors. The objective of this research is to determine the relationship between gamification elements and SHF that influence the productivity of software development teams. This research included the design of a scoring template to collect data from the experts. The importance was calculated using the Simple Additive Weighting (SAW) method as a tool framed in decision theory. Three criteria were considered: cumulative score, matches in inclusion, and values. The relationships of importance serve as a reference value in designing gamification strategies that promote improved productivity. It extends the path toward analyzing the effect of gamification on the productivity of software development. This relationship facilitates designing and implementing gamification strategies to improve productivity.

## Introduction

Gamification is increasingly used in different contexts of society, including business, health, education, commerce, and software development [1]. Specifically, it fosters commitment, motivation, and collaboration among people in a context that is not entertainment [2,3]. Simultaneously, it seeks to support the development of human factors associated with the different contexts in which it is implemented, motivating users to complete work activities and influencing their behavior.

The software development process is considered a set of activities centered around people. Therefore, the SHF have a high level of influence on this process [4,5]. These factors are considered key aspects of the success of software development projects. Despite this, some studies around empirical work and research on this topic are working on design techniques, methods, or processes. However, only some consider the social and human dimensions of software development [6–9].

Moreover, software development productivity is a topic of interest for software organizations. Different factors that influence productivity have been identified, such as technical type, organizational type, those at the project level, and those associated with SHF [10–15]. In this context, the need arises to identify SHF that influences the productivity of software development teams and propose new improvement strategies supported by gamification. Thus, it is essential to determine how gamification elements are related to these factors to design strategies to enhance a specific SHF. Then, the research question is, what are the relationships of importance between gamification elements and SHF that influence the productivity of software development?

Consequently, this research aims to identify a relationship between the gamification elements and SHF from the experts’ scoring concerning the importance of these elements in promoting the development of certain SHFs. This study used SHFs from previous research, which identified 13 SHFs that impact software development teams’ productivity [15]. These SHFs support the proposal of improvement alternatives that support their development and positively influence productivity.

This study analyzed the criteria of gamification experts, and the data collected was analyzed using techniques framed in decision theory [16] to answer the research question. Accordingly, this paper presents the relationships of the importance of 13 SHF that influence the productivity of software development teams for each of the 24 gamification elements characterized. The results of the study involved a set of data that was analyzed using the SAW method [17,18], which makes it possible to select the best of several alternatives considering a set of criteria. In this case, the method facilitated identifying relationships between elements and SHF.

The results obtained allow us to identify high, medium, and low levels of relationships. We will link these two aspects (elements and SHF) to facilitate the design and implementation of gamification strategies that positively influence the productivity of software development teams. Moreover, such a relationship extends a new path toward analyzing the effect of gamification on software development productivity, from analyzing its effect on SHF to psychological need satisfaction.

The main contributions of the paper are fourfold. First, the paper provides the relationship between gamification elements and human factors, which is helpful for companies because they can select what elements to use to foster specific human factors. Second, this research’s findings support contexts close to gamification, such as serious games, because it provides a helpful tool to define which elements to use depending on what human factors they want to develop. Third, the results of this research complement the SHFs, which are characterized by their appropriate relation to gamification elements. Thus, companies can foster them through these recommendations and design adequate strategies based on gamification. Fourth, in the context of Industry 4.0 and according to the educational challenge, the SHFs analyzed in this study can contribute to the curricula design to promote the training process.

This article follows the following structure: the Related Work section presents the most relevant scientific literature related to the research topic. The Materials and methods section describes the methodological process that was used in this research study and the Discussion section presents an analysis of the findings from different perspectives. The Limitations and validity threats section acknowledges potential biases and constraints of our approach, addressing internal and external validity concerns. The final section synthesizes the key findings and outlines promising directions for further research..

## Related work

Before presenting our research methodology and findings, it is essential to establish the theoretical foundation and current state of knowledge in the field. This section examines existing literature across three key areas: gamification and its applications, SHF in software development teams, and the intersection of these domains. Through this review, we identify gaps in current understanding and position our research contribution within the broader academic discourse.

### Gamification

Gamification aims to incorporate game mechanics and elements in contexts that are not traditionally game. Incorporating game mechanics and elements in those contexts improves commitment, motivation, and performance among users, making the perception of their work more appealing [3].

Gamification is applied in different contexts [19]. Commerce, education, health, organizational systems, work, and innovation are some of the most outstanding. However, despite the variety of those contexts, the goal is the same, i.e., to positively influence the results at the user’s psychological and behavioral level. Therefore, gamification is applied in many fields to help encourage behavior change in people and promote desired attitudes [20]. Similarly, gamification intends to incorporate game elements to change user behavior, and some changes can generate improved commitment, motivation, and enjoyment [21].

There are some works dedicated to the literature review in the context of gamification, either as mappings or as a systematic review. In this sense, Klock et al. [22] showed an increasing number of studies related to tailored gamification between 2015 and 2017. Most of the papers were evaluative models and applied to the educational context. Additionally, a few papers reported methods that foster tailored gamification. Likewise, Krath, Schürmann, and Von Korflesch [23] carried out a systematic review and identified more than 100 different theoretical foundations “used to design and evaluate gamified interventions.” These theories are included or related among them. From the theoretical foundations found, they were able to draw basic theoretical principles that made it possible to explain how gamification works and share parallels with several design guidelines for successful gamification. Klock et al. [22] and Krath et al. [23] agreed about gamification elements in their review of the literature, such as badges, challenges, customization, and feedback, among others. In gamification, there is no magic formula to set the goals; for this reason, it is important to consider the user’s characteristics.

### SHF in software development teams

On the other hand, developing a software product is characterized as a social activity governed by human-centered tasks in which SHF plays an important role [5,24]. Tasks related to software development require working teams with capabilities associated with collaboration and cooperation [25–27]. In turn, completing these tasks requires skills in social interaction among the team members to share information accurately, discuss ideas, and make timely decisions. Thus, SHF can influence the productivity of software development teams and the success of projects.

The importance of SHF engendered an interest in studying SHF that influences software development productivity. Machuca-Villegas et al. [15] considered a psychological and software engineering perspective for analyzing some SHF. As a result of this study, 13 SHFs were obtained: communication, collaboration, commitment, motivation, work satisfaction, leadership, innovation (creativity), emotional intelligence, autonomy, empathy and interpersonal relationship, team cohesion, capabilities and experiences in the software development process, capabilities and experiences in software project management.

In summary, although the influence of gamification on participant behavior has a long history, it is an emerging topic of considerable research in recent years. For this reason, relevant motivation exists in the investigating topics such as user engagement [28] and intrinsic motivation, the need to be competent [29], and participant performance and its effect on psychological needs based on self-determination theory [30], among others [31], as well as the influence of gamification on the SHF of software development teams [32,33].

### Gamification and SHF in software development teams

In this line, we found studies that identified the influence of gamification elements on different psychological needs related to Software Engineering. For instance, Lombriser, Dalpiaz, Lucassen, and Brinkkemper [34] proposed a gamification model to elicit requirements in software development projects. To evaluate this model, they implemented a gamified web platform that includes several gamification elements to affect broad psychological needs. One of the results of this experience was the positive effect of the leaderboard gamification element on the competition of the software development team. Likewise, Chow and Huang [35] described a model that integrates concepts associated with Social Software Engineering, the Capability Maturity Model, gamification, and psychology. In this model, they enunciate the effect that gamification elements can generate on a player’s emotions. This study also relies on Maslow’s hierarchy model to explain the influence of gamification on the satisfaction of human needs.

Similarly, Sailer et al. [30] investigated the influence of gamification on the fulfillment of basic psychological needs, taking self-determination theory as a reference. The study’s results revealed the use of gamification elements to address specific psychological needs. For example, the gamification elements called badges, leaderboards, and performance graphs encouraged competence and autonomy. The avatars, meaningful stories, and teammate elements strengthened relatedness.

The work of Xi and Hamari [36] also studied the effect of gamification features (immersion, achievement, and social-related features) on the fulfillment of basic psychological needs. The results indicate that some game features were positively associated with psychological needs. Among them, immersion-related gamification features (such as avatars, roleplay mechanics, storytelling, and narrative structures) were only positively associated with autonomy. Achievement-related and social-related features were positively associated with all kinds of satisfaction.

Additionally, the literature review conducted by Barreto and Franca [37] recorded a high percentage of gamification’s positive effect on software engineers’ behavior, such as collaboration, motivation, performance, and learning.

Existing relevant studies have identified the benefits of gamification as a tool to influence participant behavior [38–42]. These studies will establish gamification as an alternative to involving SHFs that positively influence software development productivity. However, there are relevant research lines. One line of research pertains to models that facilitate the identification of suitable gamification elements for gamified design strategies. Another relevant research line is analyzing the effects on the motivation of the stakeholders and the process involved. There are few empirical studies related to determining the effect of gamification elements on the satisfaction of psychological needs and user behavior [30]. Generally, researchers report the involvement of gamification elements like points and reward systems in motivation. However, they identified challenges in designing and implementing a strategy [39,43].

Another research related to gamification was carried out to determine the impact of user orientations on the preference and perceived sense of accomplishment for different gamification designs in gamified systems [44]. This study concluded that it is possible to adopt the most suitable gamification designs for each system user and that customizing gamification can support designers in positively impacting users.

Although the studies reviewed in the literature can suggest a relationship between gamification elements and psychological needs, additional attributes related to these relationships are necessary. Such attributes facilitate the selection of the most appropriate gamification elements to incorporate during the design strategy. Therefore, we propose an importance relationship between gamification elements and SHFs. The established relationship supports selecting the most appropriate elements to guide the design of gamification strategies; hence, it expands the options, improving its design.

## Materials and methods

**Fig 1:**
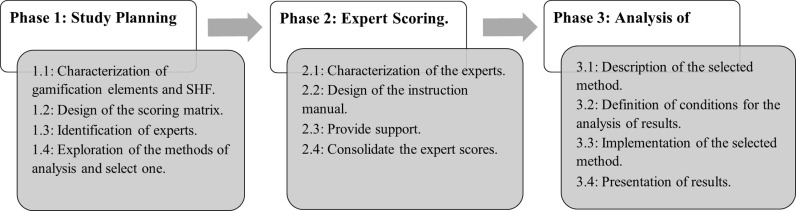
The process to establish the relationship between gamification elements and SHF. Each of the study’s phases is described in detail below.

This section describes the process followed to establish the relationship between gamification elements and SHF that influence software development productivity. The process comprises three phases: 1) the study planning and design phase, 2) the expert scoring phase, and 3) the analysis of results phase. These phases were completed over nine months, beginning in February 2020. Fig 1 describes a summary of such phases.

### Phase 1: study planning and design

The initial phase established the methodological foundation necessary to ensure systematic and rigorous research execution. This stage encompassed the characterization of gamification elements and SHF, the development of data collection instruments, the identification of domain experts, and the selection of appropriate analytical methods. These foundational activities were instrumental in establishing the validity and reliability framework for the subsequent phases of the investigation.

#### Characterization of gamification elements and SHF.

The 13 SHF were characterized [15], and based on a prior literature review, a set of gamification elements was selected. Based on the proposal by Werbach and Hunter, these gamification elements were classified into three categories: dynamics, mechanics, and components [39].

#### Design of the scoring template.

A task was designed to consolidate the information obtained from the experts. This task comprises assigning scores to establish the relationship between the gamification element and SHF. We designed a scoring matrix in which the gamification element was characterized, and the 13 SHF were listed with their respective definitions. The matrix includes space for the experts to record their scores. Table 1 presents the structure of the scoring matrix, and the sent file contains 24 sheets, each representing a gamification element.

**Table 1:** Scoring matrix.

Gamification element name	Characteristics of the gamification element	
Social factor	Definition of social factor	Score
SHF 1	SHF 1 definition	
SHF 2	SHF 2 definition	
SHF 3	SHF 3 definition	
SHF 13	SHF 13 definition	

#### Identification of experts.

Considering that the target expert population is particular and limited in availability [45], it is not convenient to use a probabilistic method to select the judges. This study searched for experts based on the information available in digital media.

The identification of experts considered their experience in Software Engineering, gamification, and psychology, as verified by the expert’s academic and research productivity, according to official databases. The research team considered their native language and verified their e-mail addresses, as it was crucial to reach out to them for participation invitations. 20 experts were identified considering these criteria: a) experience in the field, b) native language, and c) information about their contact data.

They were invited by e-mail, and 16 of them accepted. The estimated time to complete the task was two hours, which could be completed in different sessions. This task was intended to be done in person with the expert group. However, because of the emergency resulting from the pandemic, the task was adapted, and each expert could complete it individually with remote support when necessary.

#### Exploration of the methods of analysis and select one.

Decision theory, framed in operations research [16], enables the selection of the best of several alternatives, considering a set of criteria. Under the name of Multi-Criteria Decision Making (MCDM), the methods developed are classified into the following categories [46]: lexicographic ordering methods, graphical comparisons, consensus maximization comparison, additive models, and concordance methods.

In the category of additive models, also called compensatory models, the selection includes a score that reflects each alternative’s usefulness (or importance). Moreover, this category includes techniques such as SAW, Simple Multi-attribute Rating Technique (SMART), Multi-attribute Trade-Off System (MATS), Planning Assistance Through Technical Evaluation of Relevance Numbers (PATTERN), Saaty’s Analytical Hierarchy Procedure (SAHIP), or Probabilistic Linear Vector Analysis (PROLIVAN) [46].

The SAW method, framed in decision theory, was chosen because evaluations, not categorizations, were sought in the relationship between the elements of gamification and SHF. Therefore, this method uses sums of scores instead of average or mean. This method maintains the relative order of magnitude of the standardized scores by applying a linear transformation proportional to the original data [47]. Furthermore, normalized values make it easier to determine differences between compared objects [48]. It is a valuable method to select the best among several alternatives considering a set of criteria where it is frequently used [17], and it is a simple method to use [18]. This method is appropriate in this case because it restricts the criteria to positive values and requires that all criteria be maximized in nature [47]. These features ensure that SAW is a convenient method.

### Phase 2: expert scoring

The second phase centered on systematic data collection through expert evaluations. This stage implemented detailed procedures for expert characterization, instruction material design, and score consolidation. The process incorporated mechanisms to ensure data quality and consistency while maintaining standardized evaluation conditions across all participants, thereby strengthening the robustness of the collected data.

#### Characterization of the experts.

Once the experts were identified, 16 gamification experts from Mexico, Chile, Spain, and Colombia agreed to take part in the activity. Such professionals have more than five years of experience in software engineering, gamification, or psychology, as verified by postgraduate studies, research conducted in the field of gamification, and experience in the software industry.

This research did not require a formal ethics committee review as it involved collecting expert opinions through scoring matrices and did not include interventions with human subjects, sensitive personal data collection, or vulnerable populations. However, ethical research practices were followed throughout the study. The experts who contributed to this study provided informed consent via e-mail. They were informed about the purpose of the research, the voluntary nature of their participation, and how their input would be used. Written consent was not deemed necessary, as the consent was provided electronically and recorded through e-mail correspondence. Table 2 shows the experts’ profiles.

**Table 2:** Profile experts.

Expert	PhD.	Master	Undergraduate	5 years experience in Software Engineering	5 years of experience in psychology	5 years of experience in gamification
E1		X	X	X		X
E2			X	X		X
E3	X		X	X		X
E4	X		X	X		X
E5	X		X	X		X
E6	X		X	X		X
E7	X		X	X	X	X
E8	X		X	X		X
E9			X		X	
E10		x	X	X		X
E11	X		X	X		X
E12	X		X	X		X
E13			X		X	
E14		X	X	X		X
E15	X		X	X		X
E16		X	X	X		X

#### Design of the instruction manual.

Considering that the task was to be completed remotely by each selected expert, the research team designed an instruction manual to specify the procedure to be followed to fill out the Scoring template. The manual indicated the following aspects:

- Each sheet of the file corresponds to a gamification element, so each expert must fill out 24 sheets.
- The third column is to fill in the score assignment for each SHF.
- If a score is empty, then the score is equal to zero.
- Read the definition of each gamification element.
- Read the definition of each SHF.
- Make sure that the sum of the assigned scores is 100.
- Save changes to the file periodically.
- Please e-mail any concerns that may arise. We will solve them within 24 hours.

The procedure is based on the scores of experts. These experts distributed 100 points for each gamification element, depending on their experience and knowledge. We sent an e-mail to each selected expert, including the instruction manual for conducting the study and a file containing the matrices (Table 1) of the 24 selected gamification elements. Table 1 presents the structure of the file sent to each expert.

#### Provide support.

We conducted follow-ups while the experts were working on their assigned tasks, which included resolving their concerns and encouraging them to participate in the study. In most cases, the concerns raised were related to the process itself, the definitions of the gamification elements, and the SHF.

#### Consolidate the expert scores.

We used the format the experts sent as a template to consolidate and process the data. As the experts sent their responses, we consolidated the data. The complete database is available on Zenodo [49]. We verified that the sum of the scores in all cases equaled 100. The research team consolidated the data for each element and each SHF, obtaining the sum of the experts’ scores. Moreover, the number of experts who assigned a score different from zero and the number of experts who assigned the same score were consolidated.

### Phase 3: Analysis of results

The final phase focused on the systematic processing and analysis of the collected data. This stage encompassed the implementation of the SAW method, the establishment of analysis conditions, and the methodical interpretation of results. Through rigorous analytical procedures, raw data were transformed into meaningful insights regarding the relationships between gamification elements and SHF, providing a foundation for evidence-based recommendations in software development contexts.

#### Description of the selected method: SAW.

The SAW method is a multi-criteria decision-making technique that evaluates multiple alternatives based on weighted criteria, producing a single value that represents the relative importance of each alternative [50]. The SAW process for each of the 24 elements of gamification is as follows: Let ${\text{A}}_{1},{\text{A}}_{2},\ldots ,{\text{A}}_{\text{n}}$ be the set of n alternatives, which in this case corresponds to the 13 SHF previously described in Gamification and SHF actors in software development teams section. Let ${\text{C}}_{1},{\text{C}}_{2},\ldots ,{\text{C}}_{\text{m}}$ the set of m criteria that will be considered in selecting the best alternative. Three main criteria were defined for this study: cumulative score (${\text{C}}_{1})$, which represents the total sum of points assigned by all experts; matches in inclusion (${\text{C}}_{2})$ indicates how many experts considered the factor relevant by assigning it a score greater than zero; matches in value (${\text{C}}_{3})$ shows the level of consensus among experts by counting how many assigned exactly the same value.

For example, for the gamification element called emotions, an alternative is the SHF called communication. If the point assignments of the 16 experts are those shown in Table 3, then the initial score of criterion 1 is 146, which corresponds to the sum of all the scores. The starting score for criterion 2 is 14 since 14 experts gave SHF a score greater than 0, and the starting score for criterion 3 is 9 since 9 experts agreed that the score is 10.

**Table 3:** Example of calculating the criteria for an SHF in a gamification element.

E1	E2	E3	E4	E5	E6	E7	E8	E9	E10	E11	E12	E13	E14	E15	E16	${\text{C}}_{1}$	${\text{C}}_{2}$	${\text{C}}_{3}$
0	10	10	10	10	10	0	15	8	8	10	5	10	10	20	10	146	14	9

Once the point assignments of all the experts for a gamification element have been obtained, if ${\text{x}}_{\text{ij}}$ is the score of SHF i considering criterion j, then ${\text{v}}_{\text{ij}}$ is the respective normalized scoring. Each criterion ${\text{C}}_{\text{j}}$ must be assigned a weight ${\text{W}}_{\text{j}}$ so that the sum of all the weights is equal to 1 $\left(\overset{\text{m}}{\underset{\text{j}=1}{\sum }}{\text{W}}_{\text{j}}=1\right)$. Calculating the sum product of the weights of the criteria and the normalized scoring allows us to calculate the importance of each alternative in accordance with what is stated in Equation (1) [51]. That is, applying this formula allows us to quantify the importance of each SHF for a gamification element.


$${\text{I}}_{\text{i}}=\overset{\text{m}}{\underset{\text{j}=1}{\sum }}{\text{W}}_{\text{j}}{\text{*v}}_{\text{ij}}$$ (1)

- Procedure to obtain${\text{W}}_{\text{j}}$

To calculate the importance of each SHF for a gamification element, we need the weight of each criterion ${\text{W}}_{\text{j}}$. These weights are calculated only once and are obtained by finding the vector of eigenvalues [17] of the paired comparison matrix [52] used in the Analytical Hierarchy Procedure, as long as it is consistent.

The paired comparison matrix is a fundamental tool that enables systematic comparison of the relative importance between criteria. It is constructed as a square matrix where both rows and columns represent the criteria being compared. Each cell in the matrix represents how important one criterion is compared to another using a standardized scale of 1–9 (Table 4).

**Table 4:** Saaty’s fundamental scale. Source [53].

The Intensity of importance on an absolute scale	Definition	Explanation
1	Equal importance.	Two activities contribute equally to the objective.
3	Moderate importance of one over another.	Experience and judgment strongly favor one activity over another.
5	Essential or strong importance.	Experience and judgment strongly favor one activity over another.
7	Very strong importance.	Activity is strongly favored, and its dominance is demonstrated in practice.
9	Extreme importance.	The evidence favoring one activity over another is of the highest possible order of affirmation.
2, 4, 6, 8	Intermediate values between the two adjacent judgments.	When compromise is needed.
Reciprocals	If activity *i* has one of the above numbers assigned to it when compared with activity *i*, then *i* has the reciprocal value when compared with what *i* has.	
Ratios	Ratios arising from the scale.	If consistency were to be forced by obtaining n numeral values to span the matrix.

The research team defines this matrix by determining the importance of a criterion considering each of the other criteria, referencing the scale proposed by Saaty [53]. Thus, the paired comparison matrix is a matrix of size m×m, the main diagonal of which has a valuation equal to 1. The others are assigned considering whether criterion ${\text{C}}_{\text{i}}$ has an importance equal to h regarding criterion Cj, in which case the importance of criterion ${\text{C}}_{\text{j}}$ regarding criterion ${\text{C}}_{\text{i}}$ will be equal to 1/h.

Because the paired comparison matrix must be consistent, the consistency radius (CR) must be calculated. The consistency ratio is a critical measure that verifies the logical coherence of all pairwise comparisons made in the matrix. It helps detect contradictions or inconsistencies in the judgments. The CR helps identify whether such logical relationships are maintained throughout all comparisons.

The calculation of CR involves 8 steps described in [52]: (1) obtain normalized matrix, (2) obtain the average vector, (3) obtain total row vector, (4) obtain quotient vector, (5) calculate ${\text{λ}}_{\text{max}}$, (6) calculate the inconsistency index CI, (7) calculate the consistency radius CR, and (8) obtain the weights for each criterion.

If CR is less than 0.1, the matrix is consistent, and the vector of eigenvalues can be calculated. These values are the weights of each criterion ${\text{W}}_{\text{j}}$ that will determine the importance of each alternative. If CR is more significant than 0.1, the matrix is inconsistent; therefore, the value assignments in the paired criteria comparison matrix must be reviewed and reconsidered.

- Procedure to obtain${\text{v}}_{\text{ij}}$

Once the weights of each criterion have been calculated according to the procedure described, it is necessary to calculate the normalized scores ${\text{v}}_{\text{ij}}$ for each gamification element so that the evaluations of each criterion are comparable. This normalized score is based on the initial score that is assigned to each of the alternatives ${\text{x}}_{\text{ij}}$ and is generally presented in matrix form (Table 5). Returning to the example of the gamification element called emotions presented in Table 3, if communication is alternative 1, then ${\text{x}}_{11}$ is 146, ${\text{x}}_{12}$ is 14, and ${\text{x}}_{13}$ is 9.

**Table 5:** Initial alternatives scoring matrix.

	Criterion 1 -$C_{1}$	Criterion 2 -$C_{2}$	…	Criterion m -$C_{m}$
**Alternative 1 -** $A_{1}$	${\text{x}}_{11}$	${\text{x}}_{12}$	…	${\text{x}}_{1\text{m}}$
**Alternative 2 -** $A_{2}$	${\text{x}}_{21}$	${\text{x}}_{22}$	…	${\text{x}}_{2\text{m}}$
**…**	…	…	…	…
**Alternative n -** $A_{n}$	${\text{x}}_{\text{n}1}$	${\text{x}}_{\text{n}2}$	…	${\text{x}}_{\text{nm}}$

Depending on the person performing the analysis’s preference, they can perform normalization using one of the following two alternatives: If a higher value is better (profit) or if a lower value is better (cost) [54]. After normalization, the initial matrix is converted into the normalized alternatives scoring matrix (Table 6), which, in our case, is used to calculate the importance of each alternative. Each ${\text{v}}_{\text{ij}}$ is calculated by dividing the respective ${\text{x}}_{\text{ij}}$ between the highest value obtained for criterion j.

**Table 6:** Normalized alternatives scoring matrix.

	Criterion 1 -$C_{1}$	Criterion 2 -$C_{2}$	Criterion m -$C_{m}$
**Alternative 1** -$A_{1}$	${\text{v}}_{11}$	${\text{v}}_{12}$	${\text{v}}_{1\text{m}}$
**Alternative 2 -** $A_{2}$	${\text{v}}_{21}$	${\text{v}}_{22}$	${\text{v}}_{2\text{m}}$
**Alternative n -** $A_{n}$	${\text{v}}_{\text{n}1}$	${\text{v}}_{\text{n}2}$	${\text{v}}_{\text{nm}}$

Each ${\text{v}}_{\text{ij}}$ is calculated by dividing the respective ${\text{x}}_{\text{ij}}$ and the largest value obtained corresponding to criterion j. For example, for the gamification element Emotions and for criterion ${\text{C}}_{1}$, the normalized scores presented in Table 7 are obtained by dividing each ${\text{x}}_{\text{i}1}$ by 288. This same procedure must be repeated for the other two criteria.

**Table 7:** Example of normalized scores calculation.

SHF	1	2	3	4	5	6	7	8	9	10	11	12	13
${\text{x}}_{\text{i}1}$	146	145	129	235	133	85	83	288	48	157	90	29	32
${\text{v}}_{\text{i}1}$	0,51	0,50	0,45	0,82	0,46	0,30	0,29	1,00	0,17	0,55	0,31	0,10	0,11

#### Definition of conditions for the analysis of results.

The following conditions were defined to perform the analysis of the results, as follows:

- Reference values are used to classify the levels of the relationship.
- Classify the relationship levels as high, medium, and low.
- Color coding and range of values used to classify relationship levels

- White: low importance, with values between 0.0 and 0.39
- Orange: medium importance, with values between 0.40 and 0.70
- Green: high importance, with values between 0.71 and 1.00

- Summary and justification of highly important relationships.

#### Implementation of the SAW method.

The SAW method, described before, was used iteratively to relate the SHF that influences the productivity of software development teams. For each gamification element, the most relevant SHF (alternatives) are chosen, and hence, the SAW method was repeated as many times as the gamification elements were analyzed.

Then, to apply the SAW method, for each gamification element, there are 13 alternatives, where each alternative represents an SHF. In this way ${\text{A}}_{\text{i}}={\text{SHF}}_{\text{i}};\forall \text{i}\in \left\{1,\ldots ,13\right\}$. For each SHF, the following criteria were defined, denoted by the series $\left({\text{C}}_{\text{j}};\forall \text{j}\in \left\{1,2,3\right\}\right)$:

${\text{C}}_{1}$- Cumulative score. This criterion can have values between 0 and 1600.
${\text{C}}_{2}$- Matches in inclusion. This criterion can have values of 0, 2, 3, 4, and 5.
${\text{C}}_{3}$- Matches in value. This criterion can have values of 0, 2, 3, 4, and 5.

- Procedure to obtain${\text{W}}_{\text{j}}$

Table 8 presents the paired comparison matrix, which considers the research team’s criteria and Saaty’s fundamental scale described in Table 4.

**Table 8:** Paired comparison matrix.

	$C_{1}$	$C_{2}$	$C_{3}$
$C_{1}$	1,00	7,00	9,00
$C_{2}$	0,14	1,00	3,00
$C_{3}$	0,11	0,33	1,00

The paired comparison matrix must be consistent; hence, we proceeded to calculate the CR, following the steps described above, as follows:

Step 1: Obtain normalized matrix

**Table:** 

	${\text{C}}_{1}$	${\text{C}}_{2}$	${\text{C}}_{3}$			${\text{C}}_{1}$	${\text{C}}_{2}$	${\text{C}}_{3}$
${\text{C}}_{1}$	1.00	7.00	9.00		${\text{C}}_{1}$	0.80	0.84	0.69
${\text{C}}_{2}$	0.14	1.00	3.00	→	${\text{C}}_{2}$	0.11	0.12	0.23
${\text{C}}_{3}$	0.11	0.33	1.00		${\text{C}}_{3}$	0.09	0.04	0.08
Total	1.25	8.33	13.00		Total	1.00	1.00	1.00

Step 2: Obtain the average vector

**Table:** 

	${\text{C}}_{1}$	${\text{C}}_{2}$	${\text{C}}_{3}$	Average
${\text{C}}_{1}$	0.80	0.84	0.69	0.7766
${\text{C}}_{2}$	0.11	0.12	0.23	0.1549
${\text{C}}_{3}$	0.09	0.04	0.08	0.0685

Step 3. Obtain total row vector

$\left[\begin{array}{ccc} 1 & 7 & 9\\ 0,14 & 1 & 3\\ 0,11 & 0,33 & 1 \end{array}\right]\times \left[\begin{array}{c} 0,7766\\ 0,1549\\ 0,0685 \end{array}\right]=\left[\begin{array}{c} 2,4775\\ 0,4714\\ 0,2064 \end{array}\right]$

Step 4. Obtain quotient vector

$\begin{array}{c} 2,4775/0,7766=3,1902\\ 0,4714/0,1549=3,0431\\ 0,2064/0,0685=3,0131 \end{array}$

Step 5. Calculate${\text{λ}}_{\text{max}}$

${\text{λ}}_{\text{max}}=3,08214$

Step 6. Calculate the consistency index.

$\text{CI}=\frac{{\text{λ}}_{\text{max}}-\text{m}}{\text{m}-1}=\frac{3,08214-3}{3-1}=0,0411$

Step 7. Calculate the consistency radius.

$\text{CR}=\frac{\text{CI}}{\text{RI}}=\frac{0,0401}{0,58}=0,0708$

Step 8. The paired comparison matrix is consistent because CR is less than 0.1. A consistent matrix is essential as it indicates that the comparisons made between criteria are logically coherent and reliable. The eigenvector, also known as the priority vector, represents the final weights for each criterion. These weights are mathematically derived values that capture the relative importance of each criterion while maintaining the relationships expressed in the pairwise comparisons. By using eigenvalues, we ensure that the weights are not arbitrarily assigned but rather emerge from the systematic comparisons made in the matrix. Thus, the matrix’s vector of eigenvalues corresponds to the vector of weights for each criterion ${\text{W}}_{\text{j}}$, which is required to determine the importance of each SHF. The weight of each criterion is shown in Table 9.

**Table 9:** Criteria weights.

Criterion	${\text{C}}_{1}$	${\text{C}}_{2}$	${\text{C}}_{3}$
**Weight**	0,78539	0,14882	0,06579

- Procedure to obtain${\text{v}}_{\text{ij}}$

Once the criteria weights have been obtained, the scores are normalized, considering that the highest value is better, so each value is divided by the maximum [54]. Table 10 shows the initial scores of the 16 experts for the gamification element called emotions considering 16 experts. Table 11 shows the normalized matrix and the SAW values ordered from highest to lowest.

**Table 10:** Initial scoring matrix – emotions.

SHF	E1	E2	E3	E4	E5	E6	E7	E8	E9	E10	E11	E12	E13	E14	E15	E16	${\text{C}}_{1}$	${\text{C}}_{2}$	${\text{C}}_{3}$
${\text{SHF}}_{1}$	0	10	10	10	10	10	0	15	8	8	10	5	10	10	20	10	146	14	9
${\text{SHF}}_{2}$	0	10	15	10	10	15	10	3	7	5	15	5	5	5	20	10	145	15	5
${\text{SHF}}_{3}$	10	0	15	10	10	15	10	10	3	3	15	5	10	5	0	8	129	14	6
${\text{SHF}}_{4}$	20	20	15	10	10	20	10	10	12	20	20	20	10	20	10	8	235	16	7
${\text{SHF}}_{5}$	10	0	15	5	10	10	10	3	10	5	5	15	5	20	0	10	133	14	6
${\text{SHF}}_{6}$	0	0	0	5	5	0	5	15	4	3	5	10	5	0	20	8	85	11	5
${\text{SHF}}_{7}$	20	0	0	10	0	0	5	3	5	3	5	10	10	5	0	7	83	11	4
${\text{SHF}}_{8}$	20	50	15	10	15	20	5	15	15	20	15	15	20	15	30	8	288	16	7
${\text{SHF}}_{9}$	0	0	0	5	5	0	5	0	10	2	0	5	10	0	0	6	48	8	4
${\text{SHF}}_{10}$	10	0	15	10	15	10	10	15	14	16	5	5	10	15	0	7	157	14	5
${\text{SHF}}_{11}$	10	10	0	5	10	0	10	5	8	5	5	5	5	5	0	7	90	13	7
${\text{SHF}}_{12}$	0	0	0	3	0	0	10	3	2	5	0	0	0	0	0	6	29	6	2
${\text{SHF}}_{13}$	0	0	0	7	0	0	10	3	2	5	0	0	0	0	0	5	32	6	2

SHF_1_. Communication, SHF_2_. Collaboration, SHF_3_. Commitment, SHF_4_. Motivation, SHF_5_. Work satisfaction, SHF_6_. Leadership, SHF_7_. Innovation (creativity), SHF_8_. Emotional intelligence, SHF_9_. Autonomy, SHF_10_. Empathy and interpersonal relationships, SHF_11_. Team cohesion, SHF_12_. Capabilities and experience in the software development process, SHF_13_. Capabilities and experience in software project management.

**Table 11:** Normalized alternatives scoring matrix – emotions.

Social and human factor	${\text{C}}_{1}$	${\text{C}}_{2}$	${\text{C}}_{3}$	SAW
Emotional intelligence	1,000	1,000	0,778	0,985
Motivation	0,816	1,000	0,778	0,841
Empathy and interpersonal relationships	0,545	0,875	0,556	0,595
Communication	0,507	0,875	1,000	0,594
Collaboration	0,503	0,938	0,556	0,571
Work Satisfaction	0,462	0,875	0,667	0,537
Commitment	0,448	0,875	0,667	0,526
Team Cohesion	0,313	0,813	0,778	0,418
Leadership	0,295	0,688	0,556	0,371
Innovation	0,288	0,688	0,444	0,358
Autonomy	0,167	0,500	0,444	0,235
Capabilities and experience in managing software development projects	0,111	0,375	0,222	0,158
Capabilities and experience in the software development process	0,101	0,375	0,222	0,150

**Fig 2:**
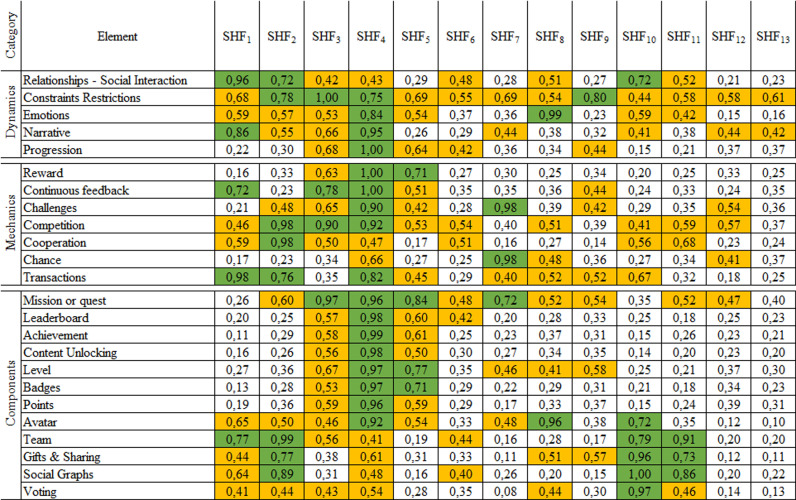
Results matrix – Relationship levels of importance. SHF_1_. Communication, SHF_2_. Collaboration, SHF_3_. Commitment, SHF_4_. Motivation, SHF_5_. Work satisfaction, SHF_6_. Leadership, SHF_7_. Innovation (creativity), SHF_8_. Emotional intelligence, SHF_9_. Autonomy, SHF_10_. Empathy and interpersonal relationships, SHF_11_. Team cohesion, SHF_12_. Capabilities and experience in the software development process, SHF_13_. Capabilities and experience in software project management.

The previous procedure was carried out iteratively for each gamification element. Hence, the results shown in Fig 2 consider the analysis conditions explained in Phase 3: Analysis of Results section. This procedure covers the fourth activity presented in Phase 3 of Fig 1.

**Fig 3:**
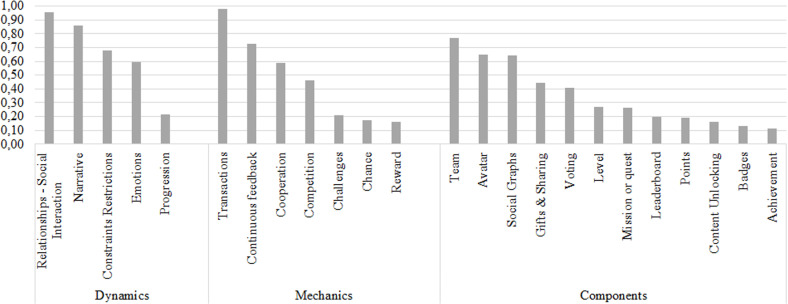
Relationship between gamification elements and the communication SHF.

Another way to analyze the results presented in Fig 2 is by SHF. Fig 3 allows us to easily identify the close relationship between the gamification elements and the factor called communication. In this case, if a software development team considers it relevant to enhance this factor, it could design a gamification strategy that includes at least the following elements: relationships - social interaction, transactions, and team, since they are the gamification elements that reported the greatest importance for this factor in the relationship established in this research.

**Fig 4:**
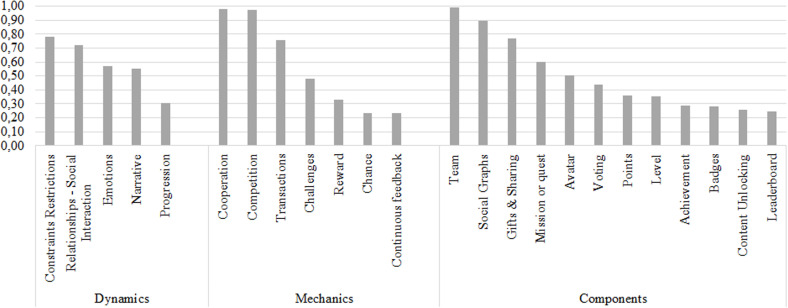
Relationship between gamification elements and the collaboration SHF.

The SHF called collaboration has importance ratings between 0.71 and 1.00 in each of the categories of gamification elements. Thus, if a software development team plans to enhance this factor, it could be guided by the relationship proposal established in this research. According to Fig 4, our results show that a gamification strategy for software development teams includes the elements of constraints, cooperation and competition, and team, and this positively affects their productivity.

**Fig 5:**
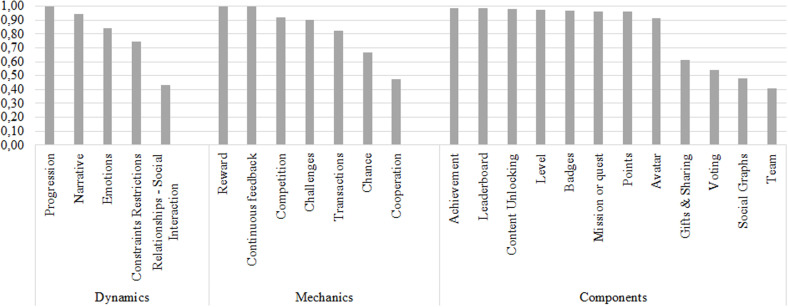
Relationship between gamification elements and the motivation SHF.

The results presented for the first two SHFs (Figs 3 and 4) can be replicated for each of the other factors. For example, suppose a software development team intends to design a strategy to influence its productivity by intervening in the motivation factor. In that case, it should analyze the results of Fig 5 to select the most important gamification elements. This research recommends that such a strategy include elements such as progression, reward or continuous feedback, and achievement or leaderboard.

## Discussion

Our findings’ analysis reveals several patterns and implications for both research and practice in software development. This section examines our results from multiple perspectives: the gamification elements themselves, the SHF they influence, and the practical implications for implementing these findings in real-world settings. We also consider how our results align with or challenge existing research in the field.

### From the perspective of gamification elements

Considering an importance threshold of 0.7, according to experts, all the gamification elements are related to at least one SHF. That means that the 24 gamification elements studied can help promote SHFs, which influences the productivity of software development teams. In addition, this study identified a relationship of importance to facilitate the choice of gamification elements in designing strategies that encourage SHFs.

Other studies suggest mappings between gamification elements and human factors. For example, [35] presents an approach to the relationships among some SHF and gamification elements by proposing a mapping between Maslow’s hierarchy of needs and some game mechanics, with a special focus on software development teams [35]. Bunchball illustrates an interaction between some game elements and basic human desires [38]. Lombriser, Dalpiaz, Lucassen, and Brinkkemper show the gamification elements used in their gamification system and their relationship with motivational factors [34] useful in software development stages such as requirement elicitation, crucial for the software development team. However, these studies use only one group of SHF and gamification elements, lacking identified relationships on the importance between them.

The gamification elements that have more SHF related to importance above 0.7 are constraints restrictions, mission or quest, and team, with each related to four SHF. These predominant elements make a difference concerning the triad of elements commonly used and recognized as PBL (points, badges, leaderboard) [39]. This finding is relevant for designers of information systems, given their particular interest in such systems offering experiences and motivations like those of games and, consequently, trying to affect user behavior [43]. These unexpected results have the potential to use other alternative elements to design innovative gamification strategies, which means that other gamification elements can be used in addition to those typically implemented in gamification experiences.

Some gamification elements can also be perceived as standing out because they favor some SHFs more than others, which indicates that they have specific psychological effects [30]. The social graphs, gifts and sharing, and team gamification elements had high-importance values associated with factors like collaboration, empathy and interpersonal relationships, and team cohesion. Similarly, social interaction, transactions and team are elements that primarily support the communication and collaboration factors. The results also show that motivation and work satisfaction can be promoted with elements like reward, mission or quest, level, and badges. In this sense, the purpose of some gamification elements is evident regarding their effect on the user. Therefore, the study of the relationship of importance confirms the application of the element and its implementation in some strategies.

### From the perspective of SHF

From the perspective of SHF, considering an importance threshold of 0.7, the experts found that all SHFs are related to at least one gamification element, except leadership, capabilities and experience in the software development process, and capabilities and experience in managing software development projects. These results demonstrate that the fundamentals of gamification provide a viable way to promote SHF, which influences the productivity of software development. The difficulty of boosting certain SHFs that did not reach a high importance value is also seen. However, it is important to conduct more empirical studies examining the context from the user’s perspective, the effect of gamification elements on SHF when implemented, and validating gamified strategies that allow their functionality to be confirmed.

Furthermore, considering an importance threshold of 0.7, the SHF that has the most related gamification elements is motivation with 17, followed by collaboration with 8, empathy and interpersonal relationships with 6, communication with 5, commitment and work satisfaction, each of them with 4. The results demonstrate that motivation continues to be the trendsetting factor in the context of gamification in many fields, including in the software development team [41], where factors such as motivation and individual behavior are crucial [55]. Studies discussing the influence of gamification elements on factors such as motivation and performance are common [29]. Performance is a relevant component of this research due to its focus on the productivity of software development teams. However, these results also highlight other SHFs that can be boosted with gamification. In this way, this research broadens the context of the application of gamification elements in relation to SHF that influence software development.

Within the 13 SHF proposed in this research and associated with the productivity of software development teams, there is a notable association with the three psychological needs proposed in self-determination theory, i.e., autonomy, relatedness, and competence. The SHF closest to these psychological needs are autonomy, empathy and interpersonal relationships, capabilities and experience in the software development process, and capabilities and experience managing software projects. This research study explores new relationship options with gamification elements in addition to those presented by Sailer et al. [30] and Xi and Hamari [36]. In turn, the SHF that can be influenced by gamification expands.

According to Sailer et al. [30], autonomy can be mapped to the avatar element as it offers players freedom of choice and to the narrative element because it can help participants experience their actions as meaningful. However, their findings suggest that the narrative element may have a positive effect on autonomy, which is not the case with the avatar element. Similarly, our research also found a relationship value between the autonomy SHF and the avatar and narrative elements; however, the importance values were low.

All the importance values of the motivation factor are at the high and medium levels, and none at the low level. These results confirm that motivation is an SHF considered influential in the productivity of software development teams and is the most predominant factor in gamification.

### Considering the importance of relationship

It is possible to find weighted scores equal to 1 when the values of the three criteria for an SHF coincide with the maximum values of the 13 SHF for an element of gamification. Therefore, most of the experts agreed with that relationship. Consequently, it was possible to obtain relationships with values of importance equal to 1, for instance, the commitment factor and the constraints restrictions element.

The SHF leadership, innovation, team cohesion, capabilities and experience in the software development process, and capabilities and experience in managing software development projects have an importance below 0.7 in the gamification elements that comprise the dynamics category. Moreover, leadership, emotional intelligence, autonomy, empathy and interpersonal relationships, team cohesion, capabilities and experience in the software development process, and capabilities and experience managing software development projects are SHF have importance thresholds below 0.7 in the gamification elements in the mechanics category. Leadership, autonomy, capabilities and experience in the software development process, and capabilities and experience managing software development projects are SHF that have an importance below 0.7 in the gamification elements in the components category. These results suggest that some SHFs fit more readily into a specific element category based on their role. This result infers a relevant finding for this work because these elements can be key triggers that affect the productivity of software development teams based on gamification strategies.

The results also identify the best relationship alternatives between gamification elements and SHF, which are critical to software development team productivity. Thus, Table 12 includes a brief definition of both the SHF and the gamification elements studied in this work to facilitate their understanding in the context of software development teams. In the relation relevance column, the study’s findings are described and supported by previous research on using gamification elements or the definition of SHF. In this way, this work strengthens and consolidates the identified relationship and broadens the definition of the gamification element towards developing these factors for the benefit of the software development team and components relevant to their productivity and performance. Leadership, capabilities and experience in the software development process, and capabilities and experience managing software development projects, are SHF do not have high-importance values; hence, values higher than 0.5 will be considered the best alternatives.

**Table 12:** The best alternatives for the relationship between the gamification elements and SHF.

SHF	Gamification Element/Category and description	Value	Relation relevance
Communication: a method that allows a human to link with another human [56]. In the work teams, the forms of communication ease the adequate flux of information, promoting dynamics that influx the outcome of software development projects[57,58].	Transactions/Mechanics: Trade between players, directly or through intermediaries), like the market to buy and sell goods [39].	0.98	There is a strong relationship between the factor called communication and the element called transactions. That may be because the transactions element requires establishing means of communication between the players to carry out any type of negotiation between them [59]. Therefore, this means that the user is within the experience and that it becomes a means of communication support, considering that communication mediates all human interaction, allowing the exchange of ideas, information, and emotions [60]. Similarly, this element can help players find opportunities for meaningful choices and relationships [36].
Commitment is the level of responsibility a team member is willing to assume in his/her tasks within the work team. In the same way, the team is responsible for the project goals [56].	Constraints Restrictions/Dynamics: established rules or restrictions that govern the gamified experience or the game [59].	1.00	Commitment corresponds to the responsibility that a subject accepts for their assigned tasks within their working team, just as the working team is engaged with the proposed objectives set within the project [15]. In this sense, it is related to the element called constraints restrictions to the extent that it seeks to attract user attention in the game and is committed to activities in the gamified experience. The restrictions give meaning to the different options that may be offered to the user, even when one would think that giving more freedom to the user would bring more enjoyment to the experience [59].
Leadership: Leadership is the capacity that some people have to influence others inside the team focused on achieving goals and objectives [61].		0.55	Leadership is a SHF that shows the influence capacity that some people have within the working team aimed at achieving their goals and objectives [59]. That is related to the element called constraints restrictions, which constitute the established rules or restrictions that govern the gamified experience. Then, using this element, limits and obstacles are established that allow the leadership of a player to manifest in front of the other participants, make decisions, and demonstrate their capabilities.
Autonomy: the capacity to make workable decisions independently from management. It directly relates to the liberty the employee and the team have to make decisions related to the project and its tasks [62].		0.80	The element called constraints restrictions represents the rules or restrictions in the game; they give meaning to the different options that can be offered to the user [59]. In this way, the player’s decision-making and competitiveness would be stimulated, making them put their skills and abilities to the test, supporting the need to be autonomous when it comes to self-efficacy [63]. In turn, this gamification element can help the participant in aspects related to autonomy, including deciding what to work on, how to solve problems, and how to adapt their work [15].
Capabilities and experience in the software development process: This includes knowledge and experience in the analysis, design, and development of a software product according to each team member’s goal.		0.58	The element called constraints can attract the attention of the game participants and motivate competitiveness among them, leading to the development of required skills. Consequently, the talent reflected in the skills and experiences of the software development process is encouraged.
Capabilities and experience in managing software development projects: It is the knowledge, application, abilities, tools, and techniques directed at the project activities [64].	Team/Components: a group of players working together to achieve a goal [39].	0.61	“Applying knowledge, skills, tools, and techniques to project activities to meet its requirements” [67] is related to the rules or restrictions established in the gamified experience or the game. Through these rules or restrictions, the user may be given different options that lead them to develop management skills, such as decision-making, planning, using resources, and defining the strategy to follow to achieve the goals set by the game.
Team cohesion: level of integration of its members so that all efforts are focused on the same common goal.	Team/Components: a group of players working together to achieve a goal [39].	0.91	The element called team can support team cohesion to the extent that team activities can be carried out to encourage socialization and collaboration among the players, helping them to direct their efforts towards a common goal [56].
Motivation: Motivation is inherent to every individual and has intensity, force, and duration. It varies according to the objective and determines part of human behavior [65].	Progression/Dynamics: This aspect reflects the game’s progress and refers to the player’s growth and development. People tend to become bored if the same experience is presented multiple times [39].	1.0	Progression is an element related to the growth and evolution of the players within the game [39]. Therefore, when designing a gamification strategy, variety in the proposed activities is sought, as this favors the game dynamics. Thus, it becomes a motivating element to promote the advancement of the experience and prevent participants from getting bored.
	Reward/Mechanics: Benefits are obtained by accomplishing an achievement or action [39].	1.00	Rewards motivate people. A balance between extrinsic and intrinsic rewards is recommended, as extrinsic motivators can displace intrinsic motivators [39]. When motivation is extrinsic, it is related to the conditions that surround individuals when they work, which, in this case, are the benefits obtained from their achievements [15]. When motivation is intrinsic, it means that people do things because they like them and they matter. This includes feelings of fulfillment, growth, and professional recognition manifested in tasks that constitute a challenge [15].
	Continuous Feedback/Mechanics: Information about what the player is doing [59] and the result of their actions in real-time [66]	1.00	The feedback within a gamified experience becomes a motivating element for its participants. This can be illustrated by providing information on game progress [22], points earned, and user performance [39]. Similarly, the information provided motivates and helps players see a participant’s productivity and, therefore, helps provide self-evaluation of their work [67].
Work satisfaction is determined by the discrepancy between what one wants and what one has in the job [68].	Mission or Quest/Components: It is a set of challenges that the player must overcome. A mission organizes the player’s effort [66], and a quest represents an assignment or task.	0.84	The mission or quest element refers to the obstacles that the participant should face; its purpose is to stimulate the user’s competitiveness. This is combined with work satisfaction because this factor is based on the challenging and stimulating activities in a person’s position, which is associated with expectations, recognition, and relationships with colleagues and superiors, among other things [15].
Innovation: related to the creativity needed to elaborate on new, different, and valuable through experiences and know-how [56].	Challenges/Mechanics: Tasks, challenges, or puzzles that need the effort to be solved and may take time, specific skills, or creativity [59].	0.98	This relationship is because the element of the challenge represents tasks, puzzles, or challenges that require an effort to solve [22]. This effort may require time, specific skills, or creativity [39,59]. Similarly, the innovation factor requires creativity, which involves developing something new, different, and of a certain value, depending on the experiences and knowledge that an individual possesses [56]. Once the puzzles or challenges are overcome, the user demonstrates competence or mastery [59].
	Chance/Mechanics: Elements of randomness in the gamification design [39].	0.98	The chance element stimulates the participant’s sense of curiosity or joy by allowing random events during the gamification strategy [39]. This can help foster innovation, where creativity and the creative capacity to establish relationships with the facts or to integrate them in a different way than usual, original and innovative, can be awakened [56].
Emotional intelligence: This is associated with participants’ emotional intelligence, which is defined as an individual’s ability to identify and adequately process their emotions so that they are not dominated by them but instead in control of their behavior [69].	Emotions/Dynamics: This element integrates a set of emotional responses that are sought out in the gamification experience, such as curiosity, competitiveness, frustration, happiness, or creativity [59].	0.99	The element called emotions integrates a set of emotional responses that the gamification experience seeks to generate. Some of them may be related to playfulness or enjoyment [22] [NO_PRINTED_FORM], and others, like curiosity, competitiveness, frustration, happiness, or creativity. Therefore, it may be associated with participants’ emotional intelligence, which is defined as an individual’s ability to identify and adequately process their emotions so that they do not dominate them but instead are in control of their behavior [15]. These emotional responses can influence relationships with team members as well as conflict resolution.
Empathy and Interpersonal Relationships: This is the mental state in which an individual relates to another individual or a group that shares the same mood.	Social Graphs/Components: Structure of the social network of the game’s participants [39].	1.00	The social network represented through the social graphs element in a gamified experience can help players or participants see their friends as potential allies, competitors, or other participants within the game [39,59]. This favors empathy and interpersonal relationships, thereby identifying with the other person or group, interacting with others based on respect, and promoting good interpersonal relationships. This is in keeping with the social nature of human beings within the dynamics of socialization with their coworkers [15].

### Practical implication

Based on the above, it is necessary to remember that the gamification elements are not the game or the gamification experience itself. The success of these elements hinges on their integration into a gamification strategy, as they alone do not reveal whether the experience is enjoyable, appealing, or yields the anticipated behavioral changes [39,40]. Therefore, a relevant contribution of this research is that this approach between gamification elements and SHF is a starting point to suggest alternative solutions that should be integrated with other aspects of strategy design to ensure the success of a gamification strategy in the context of the software development team. In this context, it is crucial to design each strategy, considering the characteristics of the users or participants, specifically the software engineering work teams. This statement coincides with Klock et al. [22] and Krath et al.[23], who, from their literature reviews, concluded that there are no magic formulas or universal recipes to design effective gamification strategies.

Beyond theoretical relationships, the practical implementation of these findings requires concrete examples to guide organizations. Our research findings can be put into practice through specific implementation scenarios that demonstrate the practical application of gamification elements to improve SHF in software development teams.

For example, organizations addressing knowledge transfer challenges can implement social graphs and team elements through digital platforms that facilitate peer-to-peer learning. These implementations typically involve creating structured environments where developers share technical solutions, participate in teaching sessions, and track knowledge-sharing metrics through team dashboards.

Integrating elements of progression and continuous feedback can address project management challenges. This can be achieved through visual project roadmaps that clearly show milestone achievements, combined with daily team progress indicators and real-time feedback loops for task completion.

Organizations can implement a combination of challenges and chance elements to stimulate innovation. This approach typically involves setting up periodic innovation challenges with rotating themes, implementing random matchmaking systems to brainstorm solutions, and creating recognition systems for novel solutions.

These implementation scenarios demonstrate how organizations can translate the theoretical relationships identified in this study into practical applications with measurable results. It is important to note that each implementation must be carefully tailored to the specific organizational context and team dynamics, with clear metrics to assess success. The effectiveness of these implementations often depends on careful consideration of the organization’s culture, team composition, and specific productivity goals.

Finally, the importance values described in Table 10 facilitate defining recommendations for software organizations to design gamification strategies that support the development of SHFs that influence the software productivity of their software development teams. High values indicate a strong relationship between both aspects (elements and SHF); hence, they will be considered the priority without the other options being ruled out. The relationship established in this research is another relevant contribution because a) the relationship has scientific rigor, using the SAW method during the definition process of the relationship, and b) such relationship constitutes a guide of recommendations for selecting elements to manage SHF that influence software development productivity. Implementing this guide should involve considering the type of situation in which the SHF to be promoted is framed as well as the creativity of the team designing the strategy. Moreover, these results could help respond to gamification strategy concerns when designing, including the following:

- What gamification elements can be used with other elements based on their related SHF?
- What gamification elements belonging to each classification category can be selected to encourage an SHF?
- What gamification elements can group more than one SHF when designing a gamification strategy?
- What SHF can be grouped to develop a gamification strategy design?

## Limitations and validity threats

This section carefully examines both internal and external validity threats and the steps taken to mitigate their impact on our findings. Understanding these limitations helps contextualize our results and guides future research directions.

The main limitation of this study is related to the number of gamification experts, and even more so when they are also required to work in software engineering. Despite this limitation, the personalized call distributed for this study resulted in 16 experts (out of the 20 experts identified) who collaborated on it. While this could be considered a small number of experts, the scores assigned by each of them and the analysis carried out using the SAW method offered initial results to suggest a relationship between the 24 gamification elements and the 13 SHF.

This study has internal validity threats influencing its process and results, such as:

- An internal validity threat in this study is associated with the subjective nature of the experts’ scoring. To overcome this threat, the research team consulted more than one expert, and they used standardized forms. Furthermore, the SAW method, framed in decision theory, was defined for expert opinions. The SAW method and the criteria defined foster an objective and rigorous analysis of the weights assigned to obtain the best alternative solutions for this study.

While the weighting assigned to the criteria used is subjective insofar as it is inherent to the method, the expert valued the weights specified in Table 9; hence, the study can be replicated. Similarly, the researchers discussed these weights to make the most appropriate decision for the study.

- Researcher bias is another common internal validity threat in research. In this work, bias may be associated with interpreting the results. To mitigate that threat, support from all working team members was considered: i) a more experienced researcher in charge of conducting the SAW method and ii) three researchers in charge of reviewing the results obtained.

Moreover, in this study, it was identified following external validity threats:

- Although the research team consulted experts with experience and academic background in gamification in software engineering, the generalization of the results may be limited by their opinions. This demands that experimental studies be carried out that extend the generalization of these results by analyzing the effects of gamification elements (from the context of the participants) on the SHF that were used in this study, thereby corroborating the established relationships.
- Since the study was conducted and financed by Spanish-speaking universities, it has an inherent language limitation, so the results can only be generalized for this context.

## Conclusions and future work

This article presents the opinion of 16 experts on the importance of the relationship between 24 gamification elements and 13 SHF that influence the productivity of software development. This relationship was established based on the scores assigned by gamification experts and used to conduct a decision-making analysis with multiple criteria using additive models with the help of the SAW method. This method facilitated the selection of the best relationship alternatives between gamification elements and SHF.

This study established a relationship between each of the gamification elements and SHF. Within these relationships, there is a trend of more association between some gamification elements and SHF than that observed in others. This suggests a specialty of gamification elements considering factor development. For example, the gamification element called team stands out as supporting SHF, which encourages teamwork.

While motivation continues to be one of the most predominant SHFs in managing gamification, this study led to new SHF options that can be promoted with gamification elements. To illustrate this, we can cite emotional intelligence, such as an SHF, which has importance values higher than 0.7 with the emotions and avatar elements.

New alternatives for appropriate gamification elements are emerging to support SHF, including constraints restrictions and avatar. In this sense, gamified strategies with these alternative elements could be proposed, such that the risk of falling into “pointsification” can be mitigated [39].

The importance values that relate gamification elements to SHF can be used as reference values to design gamification strategies that support these types of factors. In the context of this study, the values will be used to suggest a recommendation regarding more appropriate gamification elements to promote SHF when a strong relationship exists between them.

Given that the results obtained in this study constitute a guide for the design of gamified strategies, then a line of future work comprises the design of empirical studies that enable the evaluation of the real impact of gamification elements to enhance SHF. In this perspective, implementing a quasi-experiment is presented as a favorable way to achieve this purpose [70] This quasi-experiment describes Gamifik, a gamification strategy to foster knowledge transfer in software development teams by promoting collaboration in an academic context.

The analysis of the relationship between gamification elements and SHFs generates a future line of research focused on implementing gamified experiences that incorporate this type of relationship. Therefore, it seeks to propose designing and implementing gamification strategies that promote one or more SHFs that are supported by the elements recommended in the relationship. That is one way to validate whether this relationship is truly viable. Given that the language of the experts is one of the study’s identified limitations, the proposal suggests replicating the study with experts from other countries to compare the concordance among opinions and the potential for generalizing results.

Additionally, the results generated by the judges can be applied when assessing the relationship between the elements of gamification and the SHF in the educational context, choosing the best combination that contributes to enhancing academic performance, in which the elements of gamification and the SHF can be identified. These elements are most suitable for the development of students’ skills at different educational levels. In other words, gamification strategies can be applied to enhance learning by having this range of possibilities for gamification and SHF elements.

One line of future work is to explore the influence of gamification strategies in improving employment performance. Analyze the use of gamification strategies supported by SHF in the workplace to determine how these can improve performance at work and strengthen the skills necessary to work as a team. Also, motivate the software development team using gamification elements in the development of their activities, seeking to strengthen key SHF in a team, such as motivation, commitment, teamwork, and collaboration.

Finally, other potential areas for future research may focus on deepening the understanding of gamification elements that contribute to enhancing different SHF and exploring the design of 3D web applications that involve creating educational games to support emotional development in different contexts, both work-related and educational.

Although this research focused on software development teams, the relationships established between gamification elements and social and human factors have potential applications in several fields. The methodology and findings could be adapted to improve productivity and team dynamics in other disciplines where there is high interaction between team members, such as education, research, or medicine. For example, the relationship between gamification elements and factors such as motivation, collaboration, and emotional intelligence could be particularly valuable in coordinating medical teams or managing creative projects.

The systematic approach to combining gamification elements with specific human factors could facilitate the design of strategies during corporate training or professional development programs. This transferability is especially relevant as organizations across industries increasingly recognize the importance of human factors in team performance and seek innovative ways to improve workplace dynamics through gamification.
